# Individual- and population-level associations of mental disorders with intentional self-harm

**DOI:** 10.1192/j.eurpsy.2026.10182

**Published:** 2026-03-10

**Authors:** Philippe Mortier, Matilde Francisco, Itxaso Alayo, Laura Ballester, Juan Francisco Martínez-Cerdá, Montserrat López, Ana Portillo-Van Diest, Diego Palao, Víctor Pérez-Solà, Lars Mehlum, Ronald C. Kessler, Oleguer Plana-Ripoll, Gemma Vilagut, Jordi Alonso

**Affiliations:** 1 Hospital del Mar Research Institute, Barcelona, Spain; 2 Centro de Investigación Biomédica en Red de Epidemiología y Salud Pública (CIBERESP), Instituto de Salud Carlos III (ISCIII), Madrid, Spain; 3 Biosistemak Institute for Health Systems Research, Bilbao, Bizkaia, Spain; 4 Red de Investigación en Cronicidad, Atención Primaria y Promoción de la Salud (RICAPPS-RICORS), Instituto de Salud Carlos III (ISCIII), Madrid, Spain; 5 Agency for Health Quality and Assessment of Catalonia (AQuAS), Barcelona, Spain; 6 Department of Medicine and Life Sciences, Universitat Pompeu Fabra (UPF), Barcelona, Spain; 7 Department of Mental Health, Hospital Universitari Parc Taulí, Sabadell, Spain; 8 Institut d’Investigació i Innovació Parc Taulí (I3PT), Sabadell, Spain; 9 Centro de Investigación Biomédica en Red de Salud Mental (CIBERSAM), Instituto de Salud Carlos III (ISCIII), Madrid, Spain; 10 Department of Psychiatry and Forensic Medicine, Universitat Autònoma de Barcelona, Bellaterra, Spain; 11 Institut de Salut Mental, Hospital del Mar, Barcelona, Spain; 12 National Centre for Suicide Research and Prevention, Institute of Clinical Medicine, University of Oslo, Oslo, Norway; 13 Department of Health Care Policy, Harvard Medical School, Boston, MA, USA; 14 Department of Clinical Epidemiology, Aarhus University and Aarhus University Hospital, Aarhus, Denmark; 15 National Centre for Register-based Research, Department of Public Health, Aarhus University, Aarhus, Denmark

**Keywords:** cohort studies, mental disorders, population attributable fraction, self-injurious behaviour, suicide

## Abstract

**Background:**

Registry-based studies can inform suicide prevention by identifying mental disorders with the highest risk. Previous studies focused on severe disorders and suicide, with limited data on non-lethal self-harm or population impact. We quantified individual- and population-level associations of 32 mental disorders with non-lethal intentional self-harm (NLISH) and suicide.

**Methods:**

Registry-based cohort study representative for all residents of Catalonia (Spain) aged ≥10 years (2014–2019; *n* = 645,571). Cause-specific Cox models estimated individual (hazard ratios [HRs]) and population-level (population attributable fractions [PAFs]) associations with NLISH and suicide, stratified by sex and adjusted for age, socioeconomic status, and nationality.

**Results:**

Individual-level associations with NLISH were strongest for borderline personality disorder (BPD; females HR = 26.9 [95%CI 24.9–29.0]; males HR = 18.9 [95%CI 16.7–21.4]). Associations with suicide were strongest for BPD in females (HR = 40.9 [95%CI 28.5–58.8]) and obsessive-compulsive disorder in males (HR = 17.4 [95%CI 5.3–56.5]). Associations with suicide were stronger among females, and those aged 10–44 across mood, substance use, dissociative, borderline personality, and psychotic disorders. Substantial proportions of outcomes were associated with common disorders: depressive episodes (PAFs 29.8–49.8%), substance use disorders (PAFs 25.1–48.7%), mixed anxiety-depressive disorders (PAFs 19.7–53.2%), and adjustment disorders (PAFs 10.6–44.6%).

**Conclusions:**

Depressive, anxiety, adjustment, and substance use disorders are associated with large shares of self-harm and suicide, whereas BPD confers particularly high individual risk. Our findings support multilevel prevention strategies, especially among young people, including improved risk assessment, collaborative care, and timely access to specialized interventions.

## Introduction

Mental disorder is one of the strongest suicide risk factors [1–9] and registry-based studies leveraging healthcare data can identify those mental disorders most strongly associated with suicide [10]. Studies from Denmark [3], Sweden [11], and Taiwan [12] found relative risks 9.5–14.3 and absolute risks 6.3–6.9% for schizophrenia, mood, and substance use disorders. Existing research often focused on severe disorders such as schizophrenia or bipolar disorder [13–15], a narrow subset of mental disorders [12, 15–17], or broad diagnostic categories [3]. They often rely on inpatient data, excluding milder cases treated in outpatient or primary care settings [13, 14, 18], potentially overestimating risk [1, 19]. A critical limitation is the scarcity of studies that also include non-lethal intentional self-harm (NLISH) as primary outcome [20, 21]. NLISH is highly associated with personality disorders, especially borderline personality disorder (BPD) [22], and functions as an important warning sign for future suicide. Another gap is limited research on population-level impact of mental disorders on self-harm [23, 24]. While conventional measures of association (e.g., hazard ratios [HRs]) describe relative differences in individual-level risk, complementary population-weighted summaries (e.g., population attributable fractions [PAFs]) that jointly consider disorder prevalence and individual-level association strength may better contextualize the representation of specific mental disorders within the population distribution of non-lethal and lethal self-harm for surveillance, service planning, and suicide prevention policy.

We conducted a population-based retrospective cohort study representative of the general population of Catalonia (Spain) to examine sex- and age-specific individual- and population-level associations of mental disorders with subsequent NLISH and suicide. We included 32 mental disorders, encompassing both broad diagnostic groups and more specific disorders typically classified within broader categories.

## Methods

### Study population and cohort sampling design

**Figure 1.**
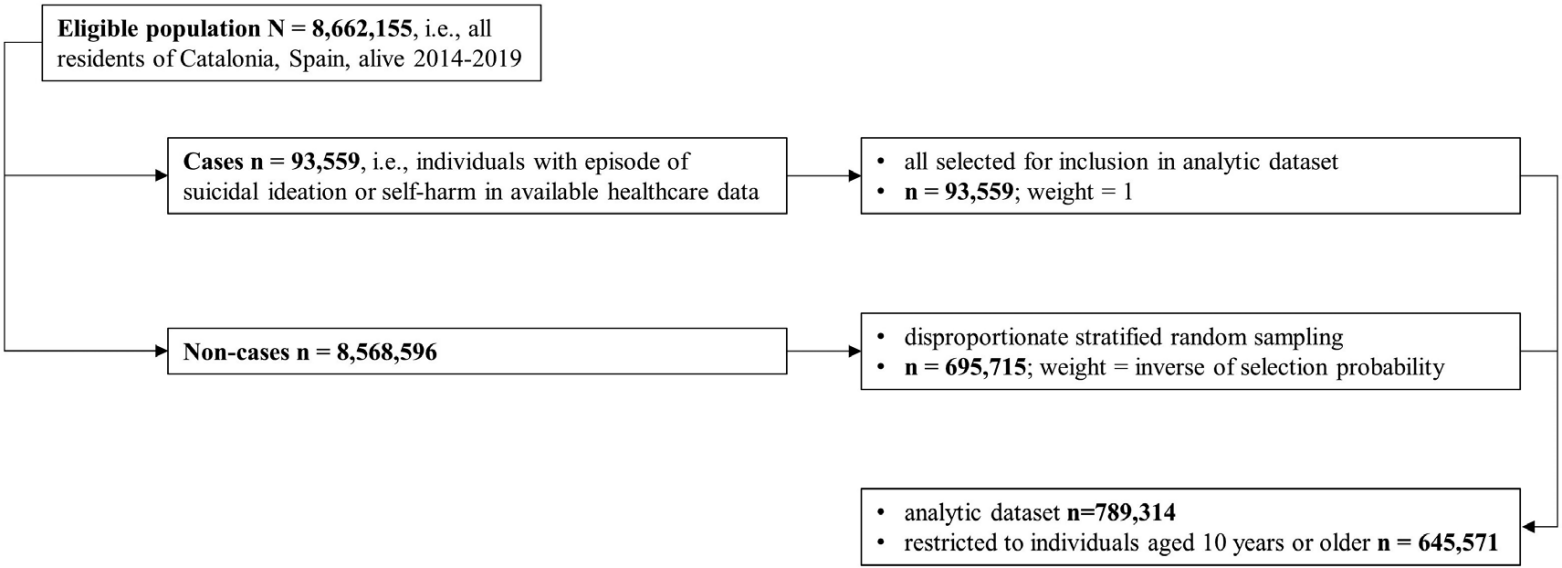
Sampling and weighting design of the study population. *Note*: See Supplementary Methods for a detailed description of the sampling and weighting procedure. Suicide deaths were not used as a sampling criterion because mortality data were not available at the time of sampling. The resulting analytic dataset is an outcome- and exposure-enriched sub-sample of the source population; inverse probability weights are therefore applied in all analyses to recover population-representative estimates for suicide, NLISH, and all other variables and associations examined.

We used the Catalan Health Service’s central population register to identify all residents of Catalonia (Spain) who were alive at any point between January 1, 2014 and December 31, 2019 (*n* = 8,662,155). A random stratified sampling design disproportionately sampling for outcome and exposure status (enrichment), similar in principle to a case-cohort design [25, 26], was applied to enhance computational efficiency in the analysis of rare outcomes and exposures while preserving population representativeness. See Figure 1 for a flow chart diagram of the sampling and weighting design of the study population. We selected all individuals with recorded suicidal ideation or self-harm events and a random subsample of non-cases. Among non-cases, we oversampled individuals with indicators of mental disorder history available in the central population register using disproportionate stratified random sampling. Inverse selection probability weights corrected for unequal selection probabilities, enabling population-representative inference. Suicide deaths were not used as a sampling criterion because mortality data were not available at the time of sample construction. Consequently, suicide deaths could occur among both sampled cases and non-cases and were incorporated analytically through inverse probability weighting. A detailed description of the sampling and weighting procedure is provided in the Supplementary Methods. All analyses were restricted to individuals aged ≥10 years in 2014. Follow-up for NLISH and suicide extended through December 31, 2019, with residents emigrating censored at date of emigration. The study protocol was approved by the Parc de Salut Mar Clinical Research Ethics Committee (2022/10325/I).

### Sources of registry data

Registry data came from the Agency for Health Quality and Assessment of Catalonia, which oversees the Public Data Analysis for Health Research and Innovation Programme [27, 28]. Using personal healthcare identification numbers, we linked registry data from four sources: (1) mortality data from Spain’s National Statistics Institute (2014–2019), including suicide coded by the International Classification of Diseases, Tenth Revision (ICD-10); (2) routinely collected electronic health record (EHR) data representing the entire Catalan public healthcare system, provided by the Catalan Health Service, including psychiatric hospitalizations (2008–2019), outpatient mental healthcare visits (2008–2019), emergency department visits (2014–2019), general hospitalizations (2007–2019), and primary care visits (2010–2019), with diagnoses coded by ICD-9-CM, ICD-10, and ICD-10-CM. EHR quality is monitored through systematic checks by health documentalists [29]; (3) clinically confirmed self-harm episodes (2014–2019) from the Catalonia Suicide Risk Code (CSRC) programme [30], which mandates psychiatric evaluation after self-harm; and (4) administrative data from the Catalan Health Service’s central population register (2014–2019), including sex, age, socioeconomic group, and nationality.

### Outcome variables

To address undercoding, NLISH was defined using two sources: (1) healthcare visits coded with ICD self-harm external cause codes (ICD-9-CM E950*–E958*, ICD-10 X60*–X84*, or ICD-10-CM T14.91*, X71*–X83*, and T36*–T65*/T71* [if the fifth or sixth character equaled 2]) in routine EHR; and (2) clinically confirmed self-harm episodes from the CSRC programme. The first NLISH event recorded in either source was coded as the outcome event. Suicide was defined as death from intentional self-harm (ICD-10 X60–X84).

### Explanatory variables and covariates

Explanatory variables included 32 mental disorder categories, created by grouping ICD-coded diagnoses from healthcare visits using the ten-level subchapter structure of ICD-10 Chapter V (F00–F99), with five categories subdivided to enhance granularity (Supplementary Table 1). Covariates were (1) sex; (2) age group (10–19, 20–44, 45–64, ≥65); (3) socioeconomic status, classified as employed or retired with contributory income above/below €18,000, or vulnerable (e.g., disability support, non-contributory pensions, minimum living income, or unemployed without benefits); and (4) nationality, grouped by country income level (high, upper-middle, lower-middle, low).

### Statistical analysis

Descriptive statistics were used to characterize the study cohort by the covariates, and sex-specific point prevalence and 95% confidence intervals were estimated for each mental disorder category. Associations of mental disorders with NLISH and suicide were examined using cause-specific Cox proportional hazards models, run separately for each of the 32 disorders, stratified by sex, and adjusted for age, socioeconomic status, and nationality (by income level). Additional models were stratified by age group. In suicide models, non-suicide deaths were competing events; in NLISH models, all-cause deaths were competing events. To establish a temporal relationship between mental disorder diagnosis and NLISH, individuals with any NLISH diagnosis prior to January 1, 2014, were excluded through a washout procedure [20], based on available pre-2014 EHR data from primary care (from 2010) and hospitalization records (general from 2007; psychiatric from 2008). Because emergency department data were unavailable before 2014, the effective washout period varied by data source and was incomplete for NLISH events recorded exclusively in emergency departments.

Mental disorders were modeled as time-varying exposures. Individuals with no mental disorder diagnosis recorded in EHR before or during follow-up (2014–2019) were considered unexposed throughout. Prevalent cases, i.e., those diagnosed before January 1, 2014, were considered exposed from the start of follow-up. Incident cases diagnosed during the follow-up period were considered unexposed until the date of first healthcare contact for the mental disorder, and exposed afterwards. Because outpatient mental healthcare visit dates were only available by calendar year, January 1 was assigned as the diagnosis date.

Mental disorders were not mutually exclusive. Individuals could be classified into more than one disorder category and could therefore contribute to multiple disorder-specific models. Each Cox model compared individuals with the disorder of interest to all other individuals in the analytic cohort, irrespective of the presence of other mental disorders. As a result, disorder-specific estimates reflect the total association of each disorder category with the outcome and are not mutually adjusted for psychiatric comorbidity. In addition, we analyzed a composite category of “any mental disorder,” defined as the presence of at least one of the 32 mental disorder categories at any point during the observation window.

To evaluate population-level associations of mental disorders with the outcomes, we calculated PAFs [31] using Miettinen’s case-based formulation [32, 33], combining the weighted prevalence of each mental disorder among cases with adjusted HRs from cause-specific Cox models. PAFs summarize the proportion of observed cases statistically attributable to an exposure under the specified model assumptions and over the observed follow-up period [33]. Although this formulation does not explicitly incorporate time or competing risks in the PAF calculation itself, it is commonly used as a summary measure in cohort studies; competing risks are addressed at the level of HR estimation [34]. Accordingly, PAFs are interpreted here as descriptive, model-based measures averaged over follow-up rather than as time-specific or causal estimands of preventable risk.

Finally, we estimated a lethality index (LI) [35] of self-harm associated with each mental disorder by dividing the suicide incidence rate by the sum of the suicide and NLISH incidence rates among individuals diagnosed with the disorder, multiplied by 100.

Statistical analysis was performed using R, version 4.4.2 (R Project for Statistical Computing), primarily using the *survey* (version 4.4-2) and *survival* (version 3.7-0) packages. All analyses were conducted within the survey design framework, accounting for inverse probability sampling weights and stratification. Point estimates, including measures of association, prevalence, incidence rates, and HRs, were accompanied by standard errors and 95% confidence intervals derived using design-based variance estimation based on Taylor series linearization (robust sandwich estimators), as implemented in the survey package. For time-to-event analyses, cause-specific Cox proportional hazards models were fitted using survey-weighted partial likelihood (survey::svycoxph), yielding design-consistent HR estimates with inference based on robust, design-based variance estimation. Confidence intervals for PAFs were derived using an analytic variance estimator combining uncertainty from both the log-HR and the weighted exposure prevalence among cases, with interval construction based on a log(1−PAF) transformation [36]. Two-sided *P* < .05 was considered significant. Analyses were conducted between October 14, 2024 and January 29, 2026.

## Results

### Study cohort descriptive statistics

The final cohort included 645,571 individuals aged ≥10 years (from 6,623,221 eligible residents). A total of 18,045 emigrated before December 31, 2019 and were lost to follow-up. During follow-up, 36,549 had one or more episodes of NLISH, and 1,118 died by suicide.

The cohort comprised 340,317 females (50.9%) and 305,254 males (49.1%). As shown in Table 1, most were aged 20–44 (38.3% in females; 41.9% in males) or 45–64 (29.1% in females; 30.1% in males), had annual contributory incomes <€18,000 (64.8% in females; 56.7% in males), and were of nationalities from high-income countries (91.4% in females; 89.2% in males).

**Table 1.** Cohort descriptive statistics (*n* = 645,571)

	Females (*n* = 340,317)		Males (*n* = 305,254)	
	*n*(uw)	%(w)	*n*(uw)	%(w)
**Age**				
10–19	40,837	10.4	44,178	11.8
20–44	119,910	38.3	117,326	41.9
45–64	100,638	29.1	91,371	30.1
65+	78,932	22.2	52,379	16.3
**Socio-economic group**				
Contributory annual income <18000 €/y	232,624	64.8	190,353	56.7
Contributory annual income >18000 €/y	84,068	31.0	96,624	40.2
Socio-economically vulnerable categories	23,590	4.1	18,263	3.1
(missing)	35	0.0	14	0.0
**Nationality (grouped according to country income levels)**				
High income	318,278	91.4	282,038	89.2
Upper middle income	10,117	4.2	7,949	3.8
Lower middle income	11,523	4.3	14,104	6.2
Low income	363	0.2	1,110	0.7
(missing)	36	0.0	53	0.0

*Note:* Weighted percentages were calculated applying inverse probability weights upon the cohort data and are representative for all individuals living in the autonomous region of Catalonia (Spain) between January 1, 2014 and December 31, 2019, aged 10 or older (*n* = 6,623,221).

Abbreviations: uw, unweighted; w, weighted.

The prevalence of any recorded mental disorder was higher among females than males (39.7% vs 30.9%, respectively; Table 2). Mixed anxiety and depressive disorders were the most common diagnosis in both sexes, affecting 20.3% of females and 11.5% of males. Females showed higher prevalence of depressive episodes (10.4% vs 4.7%), adjustment disorders (7.1% vs 4.1%), and non-organic sleep disorders (7.2% vs 4.3%). In contrast, substance use disorders were more prevalent among males (7.6% vs 2.3%).

**Table 2.** Prevalence of recorded mental disorders (*n* = 645,571)

	Females (*n* = 340,317)		Males (*n* = 305,254)	
	*n*(uw)	%(SE) (w)	*n*(uw)	%(SE) (w)
**Any mental disorder**	232,010	39.7 (0.1)	187,226	30.9 (0.1)
**Organic disorders**				
Dementia	16,979	3.2 (0.0)	10,366	1.9 (0.0)
Delirium	9,112	1.3 (0.0)	8,068	1.1 (0.0)
Other organic	8,498	1.0 (0.0)	7,322	0.8 (0.0)
**Substance use disorders**	29,882	2.3 (0.0)	69,030	7.6 (0.1)
**Psychotic disorders**	21,152	1.2 (0.0)	27,131	1.5 (0.0)
**Mood disorders**				
Bipolar	11,224	0.6 (0.0)	8,873	0.4 (0.0)
Depressive episode	85,076	10.4 (0.1)	43,341	4.7 (0.0)
Recurrent depression	21,614	1.3 (0.0)	9,955	0.6 (0.0)
Persistent mood	42,789	4.0 (0.0)	15,321	1.4 (0.0)
Other mood	4,022	0.3 (0.0)	2,572	0.2 (0.0)
**Neurotic disorders**				
Phobic anxiety	9,935	1.1 (0.0)	6,108	0.7 (0.0)
Panic	6,305	0.6 (0.0)	3,279	0.3 (0.0)
Generalized anxiety	16,987	2.0 (0.0)	9,256	1.1 (0.0)
Mixed anx-dep (incl. other/unspec.)	122,074	20.3 (0.1)	73,188	11.5 (0.1)
Obsessive-compulsive	5,122	0.4 (0.0)	5,653	0.4 (0.0)
Adjustment	66,658	7.1 (0.1)	39,638	4.1 (0.0)
Acute stress	3,946	0.4 (0.0)	2,452	0.3 (0.0)
Post-traumatic stress	2,721	0.3 (0.0)	1,198	0.1 (0.0)
Dissociative	3,865	0.3 (0.0)	1,854	0.2 (0.0)
Somatoform	9,772	1.4 (0.0)	5,140	0.8 (0.0)
**MDs assoc. w/ physio. & physical factors**				
Eating	11,476	0.9 (0.0)	2,057	0.2 (0.0)
Sleep (nonorganic)	35,221	7.2 (0.1)	22,005	4.3 (0.1)
Sexual dysfunction	955	0.1 (0.0)	13,715	3.2 (0.0)
Puerperium-related MDs (NEC)	2,814	0.2 (0.0)	/	/
Other physio-assoc. MDs	1,006	0.1 (0.0)	314	0.0 (0.0)
**Adult personality & behaviour disorders**				
Borderline PD	8,665	0.4 (0.0)	3,653	0.2 (0.0)
Other PDs	14,570	0.8 (0.0)	12,732	0.8 (0.0)
Habit/impulse	3,004	0.2 (0.0)	6,995	0.5 (0.0)
Other adult PD/behaviour	1,945	0.1 (0.0)	1,710	0.1 (0.0)
**Intellectual disability**	4,332	0.3 (0.0)	5,615	0.5 (0.0)
**Psych. developmental disorders**	3,841	0.3 (0.0)	8,352	0.7 (0.0)

*Note:* Weighted percentages were calculated applying inverse probability weights upon the cohort data and are representative for all individuals living in the autonomous region of Catalonia (Spain) between January 1, 2014 and December 31, 2019, aged 10 or older (*n* = 6,623,221).

Abbreviations: MD, mental disorder; NEC, not elsewhere classified; PD, personality disorder; SE, standard error; uw, unweighted; w, weighted.

### Associations of mental disorders with NLISH

The individual-level association of any mental disorder (i.e., any of the 32 vs. none) with NLISH was strong in both sexes (HR = 20.5 [95%CI 19.4–21.7] in females; HR = 18.4 [95%CI 17.3–19.6] in males). Corresponding PAFs were 91.6% [95%CI 90.9–92.2] and 89.2% [95%CI 88.3–90.0]. The median HRs across the 32 disorders (i.e., comparing for each disorder to the group without the disorder) were 5.9 (interquartile range [IQR] = 4.1–9.6) in females and 6.6 (IQR = 4.8–9.7) in males; corresponding PAFs were 3.4% (IQR = 2.3–13.2) and 3.6% (IQR = 2.2–13.0).

**Figure 2.**
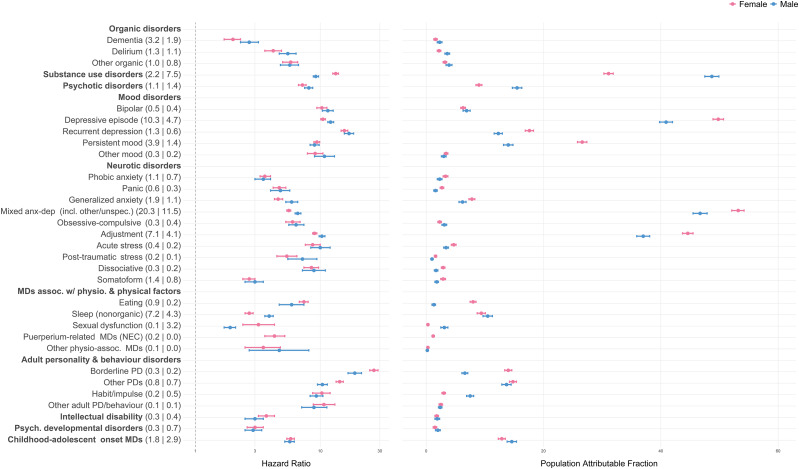
The associations of mental disorders with NLISH (*n* = 634,134). *Note:* Number in parentheses following mental disorder labels are mental disorder prevalence estimates, separate by sex (females | males). The HR and population attributable fractions are estimated for each mental disorder diagnosis separately (each time comparing to individuals without the diagnosis), using Cox proportional hazards models, adjusting for age, socio-economic status, and nationality (grouped according to country income levels). All estimates were calculated applying inverse probability weights upon the cohort data and are representative for all individuals living in the autonomous region of Catalonia (Spain) on January 1, 2014, aged 10 or older; to establish a temporal relationship between mental disorder diagnosis and NLISH, individuals with any recorded NLISH diagnosis prior to January 1, 2014, were excluded from analysis. See Supplementary Table 1 for detailed information on mental disorder diagnosis categories and corresponding ICD-9-CM, ICD-10, and ICD-10-CM diagnostic codes. Abbreviations: MD, mental disorder; NEC, not elsewhere classified; PD, personality disorder.

In both sexes, BPD showed the strongest individual-level associations with NLISH (HR = 26.9 in females; HR = 18.9 in males) followed by recurrent depression (HR = 15.6 in females; HR = 17.0 in males; Figure 2, Supplementary Table 2). Because these disorders were less prevalent, PAFs were modest (range 6.6–17.6%). In contrast, strong population-level associations with NLISH were found for depressive episodes, substance use disorders, mixed anxiety and depressive disorders (including other specified and unspecified types), and adjustment disorders, with PAFs ranging 31.1–53.2%, being highest in females with mixed anxiety and depressive disorders (PAF = 53.2%) and in males with substance use disorders (PAF = 48.7%). The individual-level associations with NLISH for depressive episodes, substance use disorders, and adjustment disorders were also considerable (HRs range 9.0–13.3 in females; 9.2–12.1 in males).

Seventeen disorders in females and 14 in males showed significant age-group variation in their associations with NLISH, most often stronger in ages 10–19 and 20–44, compared to older age groups (Supplementary Table 3). In both sexes, the overall pattern of associations was generally consistent across age groups, with the strongest associations observed for BPD (particularly in ages 10–19 [HR = 34.0 in females; HR = 28.9 in males]) and for recurrent depression (again, particularly in ages 10–19 [HR = 20.3 in females; HR = 27.7 in males]). In females, this was followed by other personality disorders (HRs range 12.3–17.2 across age groups) and substance use disorders (HRs range 9.0–17.7), and in males by bipolar disorder (HRs range 11.0–18.0) and depressive episodes (HRs range 9.0–17.1). Notably, in ages 10–19, psychotic disorders were strongly associated with NLISH in both sexes (HR = 17.4 in females; HR = 19.7 in males). In this age group, depressive episodes also showed a strong association in females (HR = 20.5) and dissociative disorders in males (HR = 17.7).

### Associations of mental disorders with suicide

Both individual- and population-level associations of any mental disorder with suicide were stronger in females (HR = 35.6 [95%CI 13.4–94.5]; PAF = 92.0% [95%CI 87.2–95.0]) than males (HR = 12.2 [95%CI 5.9–25.3]; PAF = 73.3% [95%CI 67.1–78.3]). Median HRs and PAFs were 7.7 (IQR = 4.5–12.3) and 2.7% (IQR = 1.7–12.3) in females, and 5.0 (IQR = 2.3–8.6) and 2.0% (IQR = 0.7–5.8) in males.

**Figure 3.**
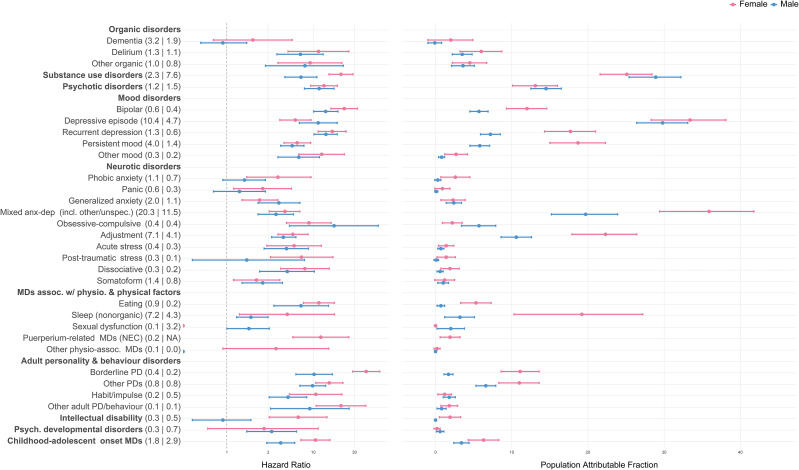
The associations of mental disorders with suicide (*n* = 645,571). *Note:* Number in parentheses following mental disorder labels are mental disorder prevalence estimates, separate by sex (females | males). The HR and population attributable fractions are estimated for each mental disorder diagnosis separately (each time comparing to individuals without the diagnosis), using Cox proportional hazards models, adjusting for age, socio-economic status, and nationality (grouped according to country income levels). All estimates were calculated applying inverse probability weights upon the cohort data and are representative for all individuals living in the autonomous region of Catalonia (Spain) on January 1, 2014, aged 10 or older. See Supplementary Table 1 for detailed information on mental disorder diagnosis categories and corresponding ICD-9-CM, ICD-10, and ICD-10-CM diagnostic codes. Abbreviations: MD, mental disorder; NEC, not elsewhere classified; PD, personality disorder.

In females, the strongest individual-level association was for BPD (HR = 40.9), followed by bipolar disorder, substance use disorders, and other disorders of adult personality and behaviour (HRs range 20.9–22.8), then recurrent depression and other personality disorders (HRs range 15.3–16.6), and psychotic disorders (HR = 13.3; Figure 3, Supplementary Table 4). In males, individual-level associations were strongest for obsessive-compulsive disorder (HR = 17.4) followed by recurrent depression and bipolar disorder (HR = 13.9–14.0), and by psychotic disorders, depressive episodes, and BPD (HRs range 10.2–11.7).

Population-level associations were strongest for depressive episodes (PAFs range 29.8–33.4%), mixed anxiety and depressive disorders (including other specified and unspecified types), especially in females (PAF = 35.9% vs 19.7% in males), substance use disorders (PAFs range 25.1–28.9%), and adjustment disorders, especially in females (PAF = 22.3% vs 10.6% in males). In females, PAFs were also considerable for non-organic sleep disorders, persistent mood disorders, and recurrent depression (PAFs range 17.7–19.2%).

Twenty-five disorders in females and 18 in males showed significant age-group variation in their associations with suicide. In females, the strongest associations were typically observed in ages 10–19 or 20–44, whereas no consistent age-related pattern was identified in males (Supplementary Table 5). When examining the overall pattern of associations between mental disorders and suicide across age groups, no consistent pattern emerged in either sex. In females, BPD was strongly associated with suicide across all age groups, especially 10–19 and 20–44 (HRs range 64.1–64.9). In females aged 10–19, even stronger associations were observed for recurrent depression (HR = 146.0), substance use disorders (HR = 113.7), persistent and other mood disorders (HR = 91.3), and dissociative disorders (HR = 70.1). In males, the strongest associations across age groups were observed for obsessive-compulsive disorder but only in those aged 45 or older (HRs range 36.1–56.9). In males aged 10–19, associations were strongest for psychotic disorders (HR = 226.5), followed by bipolar disorder (HR = 75.9), substance use disorders (HR = 56.0), recurrent depression (HR = 50.3), and BPD (HR = 33.2). In males aged 20–44, associations were strongest for depressive episodes (HR = 15.5), bipolar disorder (HR = 15.4), psychotic disorders (HR = 14.4), and recurrent depression (HR = 14.3).

### Lethality of self-harm associated with mental disorders

**Figure 4.**
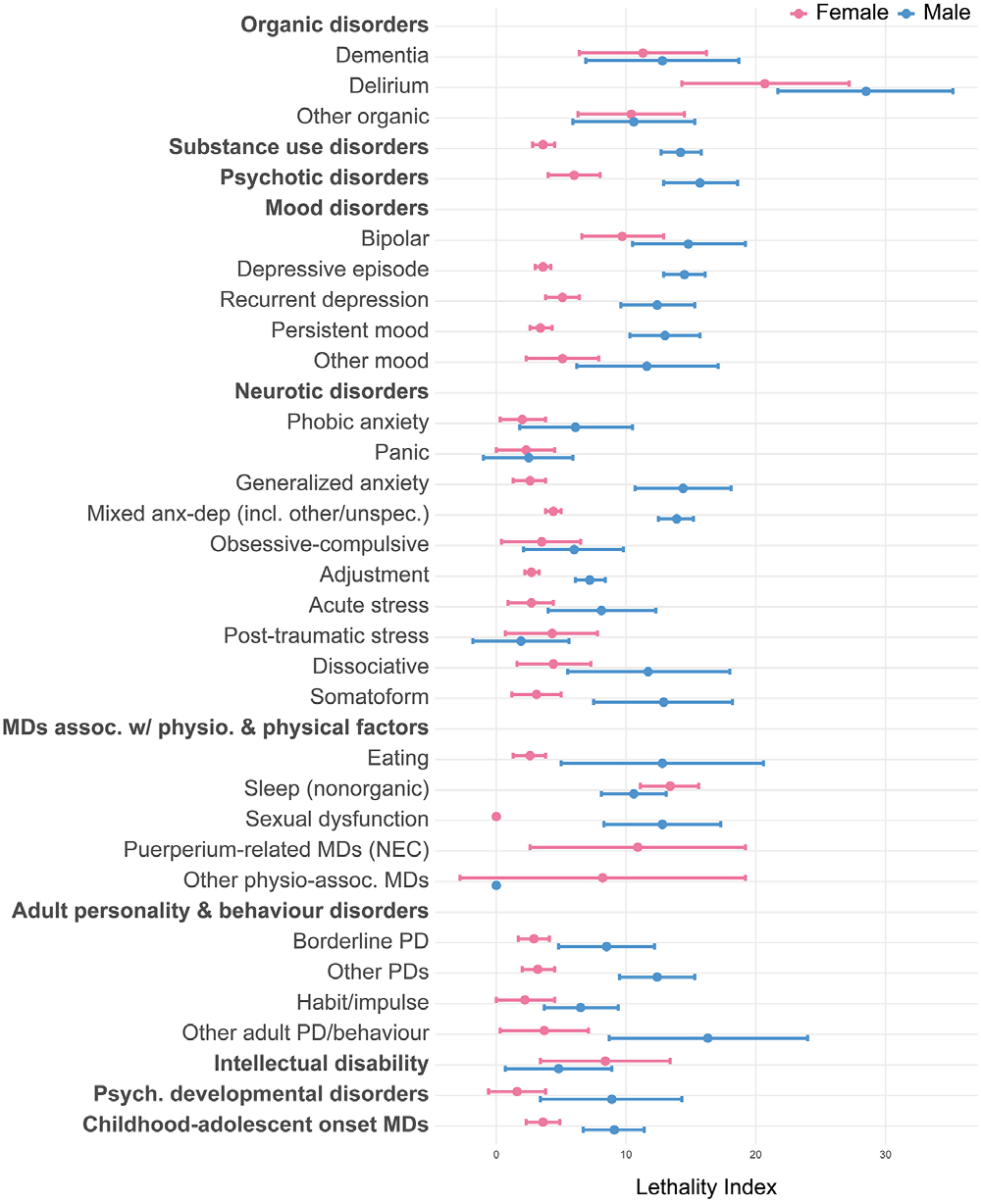
Lethality index associated with mental disorders, by sex. *Note:* The lethality index of self-harm associated with each specific mental disorder is calculated by dividing the suicide incidence rate by the sum of the suicide and NLISH incidence rates among individuals diagnosed with the specific disorder, multiplied by 100. All estimates were calculated applying inverse probability weights upon the cohort data and are representative for all individuals living in the autonomous region of Catalonia (Spain) on January 1, 2014, aged 10 or older; for the estimation of NLISH and suicide incidence rates by mental disorder, we each time excluded all individuals with any recorded diagnosis of the specific mental disorder prior to January 1, 2014 from analysis. In addition, for the estimation of NLISH incidence, we also excluded all individuals with a recorded NLISH diagnosis prior to January 1, 2014 from analysis. See Supplementary Table 1 for detailed information on mental disorder diagnosis categories and corresponding ICD-9-CM, ICD-10, and ICD-10-CM diagnostic codes. Abbreviations: MD, mental disorder; NEC, not elsewhere classified; PD, personality disorder.

The median LI of self-harm associated with mental disorders in females was 3.6 (IQR = 2.7–6.6); in males this was substantially higher, i.e., 11.7 (IQR = 7.7–13.5; Figure 4 and Supplementary Table 6). Lethality was highest for organic disorders, especially delirium (LI = 20.7 females; LI = 28.5 males). In females, this was followed by non-organic sleep disorders (LI = 13.4), mental disorders associated with the puerperium not elsewhere classified, bipolar disorder, and intellectual disability (LIs range 8.4–10.9). In males, this was followed by other disorders of adult personality and behaviour (LI = 16.3), psychotic disorders (LI = 15.7), bipolar disorder (LI = 14.8), depressive episodes (LI = 14.5), generalized anxiety disorder (LI = 14.4), and mixed anxiety and depressive disorders (LI = 13.9).

## Discussion

Our study addressed an important lack of knowledge by quantifying the population-level associations between a wide range of mental disorders and non-lethal and lethal self-harm. By presenting individual-level association estimates alongside population-level summary measures, we provide a descriptive comparison of disorders that differ in prevalence and relative association strength. A novel and important finding was that not only depression and substance use disorders [4] but also mixed anxiety and depressive disorders and adjustment disorders were associated with high proportions of NLISH (PAFs range 37.0–53.2%) and suicide (PAFs range 10.6–35.9%). These large PAFs primarily reflect the high population prevalence of these disorders, rather than exceptionally strong individual-level risks.

According to ICD-10, mixed anxiety and depressive disorder involves subthreshold symptoms of both conditions that cause significant distress yet fall short of criteria for a syndromal diagnosis. These presentations are common in primary care [28], functionally impairing, and frequently go unrecognized due to diagnostic ambiguity or somatic presentations [37]. Adjustment disorders, though often considered less severe, have been shown to be strongly associated with non-lethal self-harm among stress-related disorders [38] and are highly prevalent among emergency department and psychiatric inpatients with self-harm [39, 40]; however, research on self-harm risk in these populations remains limited. Taken together, these findings suggest that common and often under-recognized disorders represent a substantial share of the observed self-harm burden at the population level.

Importantly, the strong population-level associations observed in this study do not in themselves justify prioritizing these disorders as direct prevention targets [33, 41]. PAFs should not be interpreted as the proportion of cases that could be prevented through elimination of mental disorders, but rather as descriptive, model-based summaries of population burden under the observed mental disorder exposure structure and specified model assumptions [33, 41, 42]. Because several of these disorders are both common and highly comorbid, the estimated PAFs are not intended to represent mutually exclusive or independent effects. Approaches that explicitly adjust for psychiatric comorbidity would address a different, conditional estimand and were therefore not pursued in this analysis. Causality needs testing in robust prospective studies [43] with careful control for somatic and psychiatric comorbidity [44, 45], and other mediating and moderating factors between specific mental disorders and self-harm [46], which may all account for the associations observed in this study, and may represent more actionable targets. Even if indirect causal links are confirmed, prevention policy should ultimately be guided by the cost-effectiveness of available interventions [47].

Our study also provides robust evidence on the relationship between BPD and self-harm [48, 49], an area limited by small samples and heterogeneous study populations [50]. Since self-harm is a common feature of BPD, it was perhaps unsurprising to find BPD to be the diagnosis most strongly associated with NLISH among both females (HR = 26.9) and males (HR = 18.9) and to find low lethality of self-harm in BPD, given the recurrent course of self-harm in BPD [51]. A notable finding from our study was the exceptionally high risk of death by suicide among females with BPD (HR = 40.9), particularly among young and early middle-aged females (10–44 years; HRs range 64.1–64.9). While individual-level risks were very high, the population-level burden of BPD remained modest because of its comparatively low prevalence, especially among males. This highlights the importance of targeted interventions for individuals with BPD, including access to evidence-based psychotherapies such as dialectical behaviour therapy, structured follow-up, and crisis response plans [50].

Associations with NLISH in our study varied only modestly by sex or age, whereas associations with suicide were stronger among females, and were markedly stronger at ages 10–44 across mood, substance use, dissociative, borderline personality, and psychotic disorders, compared to older age groups. This age pattern is consistent with evidence that the suicide burden attributable to mental disorders peaks in early adulthood [4], and especially relevant given increasing rates of self-harm among young people in Spain [52]. The comparatively weaker associations in males may reflect more heterogeneous pathways less characterized by risk related to mental disorder (e.g., being single or unemployed [53], social isolation [54], legal problems [55]), suicide in the absence of mental disorder [56], lower detection of mental disorder [57], or lower help-seeking behaviour [58]. Notably, among males, risk for suicide was highest for obsessive-compulsive disorder (HR = 17.4), particularly in those aged ≥45 (HRs range 36.1–56.2). Although historically considered low risk, obsessive-compulsive disorder has recently been strongly linked to suicide in studies from Taiwan [59], Sweden [60], and Denmark [61], particularly in severe, chronic cases, likely reflecting the intense distress caused by core symptoms, independent of comorbidity [59].

This study has several limitations. First, EHR data from emergency departments, often the only point of contact for non-lethal self-harm, were only available from 2014 onwards; although a washout procedure was applied using available primary care and hospitalization registries, NLISH events occurring exclusively in emergency departments prior to 2014 could not be identified. As a result, some NLISH cases classified as incident may in fact have been prevalent. Future studies utilizing registry data spanning a larger time period will address this limitation, and will also explore whether NLISH serves as a mediator or moderator in the association of mental disorders with eventual suicide. Second, outpatient mental healthcare visit dates were only available at the year level, introducing potential immortal time bias [62] that may have led to an underestimation of associations in the Cox models. Third, the identification of mental disorders and NLISH relied on recorded diagnoses from healthcare contacts, which may be subject to underdiagnosis and misclassification, and exclude individuals who did not seek care. These limitations may have influenced prevalence estimates and, consequently, population-level measures such as PAFs. It is worth mentioning that, since public healthcare in Spain is free and highly accessible, the proportion of individuals with mental disorders who do not seek care may be relatively low [63]. Fourth, we did not investigate associations of mental disorders with repetition of NLISH, which requires specific analytic approaches [64] and was beyond the scope of this study. Lastly, while our analysis included a broad range of mental disorders, some categories were not disaggregated into specific diagnoses (e.g., substance use, psychotic, or childhood-onset disorders). Future studies will address this, and will also adjust for psychiatric and somatic comorbidity.

Our findings reinforce calls to reduce suicide mortality associated with mental disorders [65], particularly among young people. The strong population-level associations of common disorders such as depressive episodes and substance use disorders with self-harm underscore the importance of routine screening and timely referral in primary care [66, 67] and tailored prevention strategies in individuals with substance use disorders [68]. Moreover, our results emphasize the need to investigate the potential contribution of adjustment disorders and subsyndromal anxiety-depressive presentations to self-harm risk. The finding that common disorders are strongly associated with self-harm at both the individual and population level suggests that healthcare systems must be equipped to recognize and manage these conditions. Integrated collaborative care models [69, 70] coordinating efforts across primary, emergency, and mental health services offer a scalable solution. Their impact could be enhanced by data-driven risk stratification tools and clinical decision support systems [71], enabling more efficient allocation of resources. Finally, clinical strategies must also be complemented by broader policy initiatives addressing the social determinants of mental health [72].

## Supporting information

10.1192/j.eurpsy.2026.10182.sm001 Mortier et al. supplementary material Mortier et al. supplementary material

## Data Availability

The primary data, including healthcare, mortality, and administrative records, were provided by a third party, the Agency for Quality and Assessment of Catalonia (*Agència de Qualitat i Avaluació Sanitàries de Catalunya*; AQuAS), under the PADRIS (*Programa d’Analítica de Dades per a la Recerca i la Innovació en Salut*) framework. Access to these data is restricted, and must comply with PADRIS’s legal and ethical requirements. Interested parties can obtain access to the data, code, and documentation upon request, in accordance with the agreement’s provisions. The minimum dataset needed to replicate the analyses underlying this study, including the anonymized individual-level registry data, data dictionaries, and statistical code, is available upon reasonable request from the corresponding authors (Philippe Mortier: pmortier@researchmar.net; Gemma Vilagut: gvilagut@researchmar.net), provided (a) the purpose is to replicate our analysis and results without additional investigator support, (b) access is granted following approval of a brief proposal and the signing of a Data Access Agreement, and (c) the request aligns with the terms of our agreement with PADRIS/AQuAS and is approved by the PADRIS legal representative.

## Supplementary material

The supplementary material for this article can be found at http://doi.org/10.1192/j.eurpsy.2026.10182.
